# Health Literacy-Related Knowledge, Attitude, and Perceived Barriers: A Cross-sectional Study among Physicians, Pharmacists, and Nurses in Public Hospitals of Penang, Malaysia

**DOI:** 10.3389/fpubh.2017.00281

**Published:** 2017-10-19

**Authors:** Retha Rajah, Mohamed Azmi Hassali, Ching Jou Lim

**Affiliations:** ^1^Pharmacy Department, Hospital Seberang Jaya, Penang, Malaysia; ^2^Discipline of Social and Administrative Pharmacy, School of Pharmaceutical Sciences, Universiti Sains Malaysia, Penang, Malaysia

**Keywords:** health literacy, knowledge, attitude, perceived barriers, Malaysia

## Abstract

**Introduction:**

Patients’ health literacy (HL) has emerged as a critical determinant of health outcomes and becoming one of the core competencies of health-care providers. Therefore, this study aimed to assess among Malaysian physicians, pharmacists, and nurses, their HL-related knowledge, attitude, and perceived barriers, and also to determine the associated factors.

**Methods:**

A cross-sectional study design was used to enroll 600 eligible respondents using stratified sampling from 6 public hospitals in Penang, Malaysia. A validated self-administered questionnaire was used for data collection. Descriptive and inferential analysis was performed with statistical significance defined as *p* < 0.05.

**Results:**

The response rate was 87.6% with 526 questionnaires completed. Of these, 34.2% had poor knowledge, and more than half had negative attitude (51.9%) toward HL with no significant differences among physicians, pharmacists, and nurses. The majority of the respondents perceived time constraints and lack of human resources as major HL barriers. Respondents who had heard the term or concept of HL had significantly higher level of knowledge (*p* < 0.001) and more positive attitude (*p* < 0.001). While longer service years (≥11 years) significantly contribute to the higher level of HL knowledge among health-care providers (*p* = 0.028).

**Conclusion:**

The study findings supported the concern on inadequate knowledge and substantially negative attitude toward HL among study health-care providers with highest cited barriers were time constraint and human resources. Thus, efforts to improve their perspective about HL to be effective patient educators are highly advocated.

## Introduction

The importance of health literacy (HL) on influencing patients’ health outcomes is well established (1–3). There is a distinct difference between general literacy and HL. General literacy is the capacity to read, write and have basic numerical skills (4). On the contrary, HL is defined as a cognitive and social skill that determines the motivation and ability of individuals to gain access, understand, and use information in ways that promote and maintain good health (5). Education usually translates to a person’s literacy level but not necessarily their HL level. In a study conducted in two cardiac units of the University of Illinois Hospital, patients with good educational attainment were reported to have 40% likelihood of limited health literacy (LHL) (6). LHL was associated with numerous adverse health outcomes including low medication adherence (7–9), increased use of emergency care and increased risk of hospitalization (7, 10), besides unable to obtain appropriate health services and preventive health screening (11). Indeed, a systematic review documented the negative economic impact of LHL on both patient and the health system (12).

Health-care providers are obligated not only to provide health-care information and assist patients in navigating the health-care system but also carry a fundamental responsibility in meeting the health-care needs of patients with LHL. Despite the essential role of the health-care providers in building and improving patients’ HL, they were reported mostly remain unaware and lack of understanding the relevance of HL in patient care. Also, evidence consistently supported that health-care providers’ have insufficient knowledge on HL and inability to identify LHL patients (13–15). One study even reported that health providers were reluctant to conduct formal HL assessment using validated HL screening tools despite familiar with the tools (16). Similar findings were found that health providers were not fully utilizing HL assessment tools or HL strategies to enhance patient’s understanding of health information provided (13, 17). Often too health-care providers overestimate their patients’ HL level based solely on their subjective assessment (6). It is noteworthy that majority of the mentioned findings were mainly from studies conducted in advanced countries such as United States, Canada, and New Zealand.

As part of Southeast Asia countries and a developing country, Malaysia is in the progress of establishing a better health-care system to provide quality health services with a major focus on patient care. One of the key fundamental steps for the success is to improve patients’ HL. However, HL is not explicitly discussed issue in Malaysian health-care system, until in recent years the Malaysia Health Research Priority has emphasized the need for studies to evaluate HL to optimize patient care and customize health intervention to target the LHL patients. As yet, Malaysia still lacks a consolidated research to assess the HL concept from health-care provider’s perspective that will serve as valuable input to the development and implementation of future HL-based training or programs to health professionals that enable them to address the needs of LHL population appropriately. Thus, this study aimed to assess among Malaysian physicians, pharmacists, and nurses, their HL-related knowledge, attitude, and perceived barriers, and also to determine the associated factors.

## Materials and Methods

A multicenter cross-sectional survey was conducted in all six public hospitals in Penang state, Malaysia. The study period was between July and September 2016, involved three primary health professionals that are considered as front liners namely the physicians, pharmacists, and nurses. In public funded hospital settings, these health-care providers have frequent encounters with patients with chronic diseases especially those with multiple chronic conditions seeking medical assistance.

### Sample Size and Sampling Procedures

The sample size was determined using Cochrane formula (18) for continuous data with an assumption of 95% confidence interval, 5% margin error, and response rate based on prior studies for each professional group (15, 19, 20). A minimum sample size required was 200 physicians, 200 pharmacists, and 126 nurses. However, a sample of 200 health-care practitioners for each profession was undertaken given the similar nature and response rate targeted. The sample size was allocated to each facility proportioned to the total health-care providers who were working during the data collection period. A representative from each profession in the respective hospitals was identified and approached to assist in the distribution of the questionnaire forms to the potential participants and collected after 2 weeks. A brief explanation of the study that consists detail of the researchers and the contact person, research objectives, methodology, and benefit of the research was attached.

### Survey Instrument and Distribution

The data were collected using a structured questionnaire developed based on comprehensive literature review and expert opinions to assess physicians, pharmacists, and nurses’ HL-related knowledge, attitude, and perceived barriers. Questions in HL-related knowledge section used true or false with do not know answer options. One score will be awarded for a correct answer and 0 for false or do not know the answer. Attitude and perceived barriers questions were assessed using 5-point Likert-type scale responses (1 = strongly agree, 2 = agree, 3 = neutral, 4 = disagree, 5 = strongly disagree). Additional information was gathered on basic demographics (age and sex) and profession-related characteristics (the name of tertiary education/institute, years of working experience and have heard the term or concept of HL).

The questionnaire was tested for reliability and validity. The internal consistency reliability obtained for knowledge of HL (8 questions) was 0.76 measured by KR-20. Cronbach’s coefficient alpha for attitude (9 questions) and perceived barriers toward HL (10 questions) was 0.78 and 0.91, respectively, and was in acceptable range. Content validity was performed by academicians and experienced physicians, which was then followed by face validity among seven conveniently selected physicians, pharmacists, and nurses to obtain feedback on the clarity and understanding of the questions.

### Data Analysis

Descriptive statistics were used to compute continuous variables as mean and SD, and categorical variables as percentages. Correct answers for knowledge questions were calculated and summed up to give a total score. The score then was categorized into good or poor knowledge if it is equal to or above and below the mean, respectively, for each professional group (physicians, pharmacists, and nurses). Likewise, for questions in attitude section assessed with Likert scale, the mean was calculated and categorized into positive attitude if equal or above mean, and negative attitude if below mean value. χ^2^ test was used to determine the association between the categorical variables. Group differences in demographic and work-related characteristics with the mean value of knowledge and attitude were tested using one-way ANOVA or independent sample *t*-test when appropriate. All statistical analyses were performed using SPSS version 20.0 (SPSS Inc., Chicago, IL, USA). A *p*-value of <0.05 was considered to be statistically significant.

Ethical approval for conducting this research was obtained from the Medical Review and Ethics Committee, Ministry of Health Malaysia. The registration ID for the National Medical Research Register is NMRR-15-2208-28623.

## Results

The questionnaires were adequately filled and returned by 526 (87.6%) health-care providers, out of which 158 (30.0%) were physicians, 188 (35.7%) were pharmacists, and 180 (34.2%) were nurses. More female (77.8%) and local graduates (82.3%) responded to this survey. Most (85.7%) of the respondents had no more than 10 years’ work experience. Only about half (49.8%) claimed they had heard the term or concept of HL, and the finding was consistent across the three professions. The summary of basic demographic and work-related characteristics provided as Table 1.

**Table 1:** Demographics and profession characteristics of study respondents (*n* = 526).

	Designation, *n* (%)			
Demographic and profession characteristics	Physicians	Pharmacists	Nurses	Total

	158 (30.0)	188 (35.7)	180 (34.2)	526 (100.0)
**Gender**				
Male	69 (43.7)	36 (19.1)	12 (6.7)	117 (22.2)
Female	89 (56.3)	152 (80.9)	168 (93.3)	409 (77.8)

**Age, years (mean ± SD)**		**29.97 ± 6.12**		

**Age group, years**				
20–30	112 (70.9)	135 (71.8)	106 (58.9)	353 (67.1)
31–40	42 (26.6)	51 (27.1)	41 (22.7)	134 (25.5)
>41	3 (1.9)	0 (0.0)	33 (18.3)	36 (6.8)
Missing	1 (0.6)	2 (1.1)	0 (0.0)	3 (0.6)

**Working experience, years (mean ± SD)**		**5.88 ± 5.73**		

**Working experience group, years**				
<10	150 (94.9)	179 (95.2)	122 (67.8)	451 (85.7)
≥10	8 (5.1)	9 (4.8)	58 (32.2)	75 (14.3)
**Place of graduation**				
Local graduate	107 (67.7)	147 (78.2)	179 (99.4)	433 (82.3)
Oversea graduate	51 (32.3)	41 (21.8)	1 (0.6)	93 (17.7)
**Heard the term or concept of health literacy**				
Yes	69 (43.7)	98 (52.1)	95 (52.8)	262 (49.8)
No	89 (56.3)	90 (47.9)	85 (47.2)	264 (50.2)

### Knowledge, Attitude, and Perceived Barriers toward HL

Overall, more than one-third (34.2%, 180) of study respondents fall into the category of poor HL-related knowledge as shown in Table 2. Physicians (34.4%, 62) were the majority, followed by nurses (33.9%, 61) and pharmacists (31.2%, 57). The knowledge level was assessed focusing on the definition of functional HL and LHL, consequences of LHL, assessment of HL, and benefits of HL to the health-care providers. In the aspect of attitude toward HL, more than half (51.9%, 273) of the respondents were in the negative attitude score range with the higher proportion of nurses (38.8%, 106) and pharmacists (32.6%, 89). However, a comparison between the physicians, pharmacists, and nurses showed no significant differences regarding their knowledge and attitude toward HL. Table 3 outlines the factors cited as barriers toward HL among the study respondents. It is noteworthy that the highest reported were time constraint (70.0%, 368) and lack of human resources (69.6%, 366).

**Table 2:** Knowledge and attitude toward health literacy (HL) among physicians, pharmacists, and nurses.

Category (score range)	Designation				*p*-Value
	Physicians (158)	Pharmacists (188)	Nurses (180)	Total (526)	
**Knowledge of HL**					
Good (≥6)	96 (27.7)	131 (37.9)	119 (34.4)	346 (65.8)	0.22
Poor (<6)	62 (34.4)	57 (31.2)	61 (33.9)	180 (34.2)	
**Attitude toward HL**					
Positive (≥32)	80 (31.6)	99 (39.1)	74 (29.2)	253 (48.1)	0.064
Negative (<32)	78 (28.5)	89 (32.6)	106 (38.8)	273 (51.9)	

*Statistical analysis using χ^2^ test*.

**Table 3:** Factors perceived as barriers toward health literacy (HL) among respondents (*n* = 526).

Perceived barriers	Designation	*n* (%)
Time constraint	Doctors	117 (31.8)
	Pharmacists	133 (36.1)
	Nurses	118 (32.1)
	Total	368 (70.0)
Lack of human resources	Doctors	114 (31.1)
	Pharmacists	139 (38.0)
	Nurses	113 (30.9)
	Total	366 (69.6)
Lack of patients commitment toward HL intervention	Doctors	107 (30.5)
	Pharmacists	119 (33.9)
	Nurses	125 (35.6)
	Total	351 (66.7)
Lack of organizational/leadership to promote HL	Doctors	103 (29.7)
	Pharmacists	131 (37.7)
	Nurses	113 (32.6)
	Total	347 (66.0)
Lack of organizational resources to implement HL intervention	Doctors	106 (30.7)
	Pharmacists	130 (37.7)
	Nurses	109 (31.6)
	Total	345 (65.6)
Lack of patient cooperation to assess HL	Doctors	105 (30.9)
	Pharmacists	119 (35.1)
	Nurses	115 (33.9)
	Total	339 (64.4)
Lack of interest to learn about HL	Doctors	97 (31.6)
	Pharmacists	109 (35.6)
	Nurses	100 (32.7)
	Total	306 (58.2)
Lack of awareness of identifying limited health literacy patients	Doctors	92 (32.2)
	Pharmacists	93 (32.5)
	Nurses	101 (35.3)
	Total	286 (54.4)
Lack of knowledge on HL	Doctors	89 (32.2)
	Pharmacists	90 (32.6)
	Nurses	97 (35.1)
	Total	276 (52.5)

**Table 4:** Comparison of study respondents’ health literacy (HL)-related knowledge and attitude for demographics and work-related characteristics.

Variables	Knowledge		Attitude	
	Mean ± SD	*p*-Value	Mean ± SD	*p*-Value
**Gender**				
Male	5.68 ± 2.31	0.72	31.63 ± 3.86	0.24
Female	5.76 ± 2.09		31.17 ± 3.69	
**Age (years)**				
20–30	5.63 ± 2.13	0.087	31.24 ± 3.77	0.37
31–40	5.90 ± 2.07		31.07 ± 3.65	
>41	6.36 ± 1.55		32.06 ± 3.47	
**University**				
Local	5.78 ± 2.11	0.44	31.15 ± 3.73	0.11
Oversea	5.59 ± 2.05		31.84 ± 3.69	
**Working experience (years)**				
<10	5.68 ± 2.16	0.028*	31.22 ± 3.73	0.41
≥10	6.16 ± 1.67		31.60 ± 3.69	
**Heard the term or concept of HL**				
Yes	6.77 ± 1.18	<0.001*	32.12 ± 3.70	<0.001*
No	4.73 ± 2.31		30.43 ± 3.57	

*Statistical analysis using one-way ANOVA/Tukey or independent sample t-test*.

*p < 0.05 statistically significant.

### Comparison of HL-Related Knowledge and Attitude for Demographics and Work-Related Characteristics

The respondents who had heard the term or concept of HL had significantly higher mean score of knowledge (6.77 ± 1.18) compared with respondents with no prior exposure on HL (4.73 ± 2.31) (*p* < 0.001). The same pattern was noted for attitude (32.2 ± 3.70 versus 30.43 ± 3.57) (*p* < 0.001). Besides, health-care practitioners working 10 years and more (6.16 ± 1.67) had significantly higher HL-related knowledge compared with working less than 10 years (5.68 ± 2.16) (*p* = 0.028). The other factors showed no significant differences in comparison with their knowledge and attitude.

## Discussion

In the recent years, HL has increasingly been recognized as an important skill that patients require for appropriate health decisions in the fast-growing complex and overwhelming health-care system. HL is not solely the patient’s responsibility but a shared duty of both health-care practitioners and patients (21, 22). This study to the author’s best of knowledge is the first to provide an insight of the knowledge, attitude, and perceived barriers toward HL among physicians, pharmacists, and nurses serving at public hospitals in Malaysia, a developing country.

The study key finding expands the existing concern of poor knowledge of HL among the health-care providers. Physicians, pharmacists, and nurses are the immediate sources in patients seeking for health information or advice in clinical settings. These professions were ranked as one of the most trusted and preferred source (23). Unfortunately, the health-care provider’s inadequate HL knowledge can withstand as an obstacle to cater the health-related message across patients with different level of HL. One strategy to improve the health-care provider’ awareness and understanding of HL are through the introduction of HL-based continuous education programs and training in health professional’s daily practice. Indeed, the study showed health-care providers who had previous exposure to the term or concept of HL had significantly higher mean knowledge than no exposure (6.16 ± 1.67 vs. 5.68 ± 2.16, *p* = 0.028). Numerous other studies have supported the positive impact on improving providers’ knowledge and skills with the integration of HL component in the curriculum of health professional schools (24–26).

The fact that more than half (53.4%) of the study health-care providers exhibit negative attitude toward HL is quite worrying. Our study evaluated the health-care providers’ attitude broadly in terms of the importance of HL in their management and patient care, HL communication strategies, and implementation of HL programs. Somehow, other studies that have mainly focused on the attitude toward HL training showed health-care practitioners supported the need of HL training (16, 27) for effective communication in handling LHL patients. Generally, public health-care settings in Malaysia involving both health clinics and hospitals have greater patients load that seek for multiple chronic or acute conditions. The busy and stressful environment may force health-care providers to place more importance on other patient care issues that have a direct and noticeable impact on patient care. Besides, the unclear practicality of HL in the daily routine that believed may tend to lengthen the patient’s medical encounter can affect the study respondents overall attitude toward HL.

The main factors that concerned the physicians, pharmacists, and nurses were time constraints and human resources to conduct HL screening in the clinical setting. Similar findings were reported in several other studies (17, 20, 22). As HL is a new concept, the study respondents were hesitant that the screening will involve another tedious step in the existing busy clinical routine and may cause the patients to feel dissatisfied with longer waiting time. The introduction of an easy and short HL screening is pivotal approach as part of the routine patient assessment in the hospital setting. A study conducted in a non-primary care and Hispanic populated breast surgery clinic evaluating patient’s HL level using Newest Vital Sign, as part of the clinic’s vital sign assessment showed that patients well accepted HL assessment (28). When a patient diagnosed with chronic conditions, which is common in hospital setting as in our study, the requirement is for them to understand the disease and how the illness affects the body, knowing when and where to seek health advice, being able to evaluate the appropriateness of the instruction or medical advice, besides able to interpret and describe the symptoms. None of these skills and abilities required can be assumed or estimated. The health-care providers must be equipped with skills and knowledge to differentiate the varying need of information and customized communication according to patient’s HL needs. Studies have reported both subjective and objective HL assessments carry some degree of benefit in busy clinical settings whereby prioritization of a more in-depth and understandable information could be delivered to the identified patient with LHL (6, 28).

Several issues limit this study. The survey was conducted among physicians, pharmacists, and nurses and did not include another group of health-care providers. Therefore, the findings may not be representative of the entire health-care providers in Malaysia. Besides, the study adopted self-administered questionnaire. Thus, the health-care providers’ may overestimate or underestimate their actual HL-related knowledge and attitude to meet the socially acceptable behaviors. Another limitation is the cross-sectional nature of the study that represents the data at one point in time may fail to demonstrate the changes of knowledge of and attitude toward HL over time.

## Conclusion

In summary, these study findings indicate there is a great room for improvement in the HL-related knowledge, especially among physicians and nurses. A well-designed and comprehensive HL education programs integrated into the health professionals’ curriculum and continuous HL-based training during their service in public hospitals are warranted. Besides, considerable emphasis should be placed on creating a more positive attitude among physicians, pharmacists, and nurses with establishing the importance and practicality of HL in their daily clinical routine. Future efforts should aim at the adaptation of objective HL screening as part of the patient-centered approach in Malaysia public hospitals to improve the care and health outcome, especially LHL population.

## Ethics Statement

Ethical approval for conducting this research was obtained from the Medical Review and Ethics Committee, Ministry of Health Malaysia. The registration ID for the National Medical Research Register is NMRR-15-2208-28623.

## Author Contributions

RR conceptualized and designed the study, conducted data collection and analyses, and drafted the initial manuscript. MAH and CJL conceptualized and designed the study and reviewed and revised the manuscript. All contributors approved the final manuscript as submitted. All the authors state that they had complete access to the study data that support the publication.

## Conflict of Interest Statement

The authors declare that the research was conducted in the absence of any commercial or financial relationships that could be construed as a potential conflict of interest.
